# ﻿Two new species of *Acanthobothrium* Blanchard, 1848 (Cestoda, Onchoproteocephalidea) from *Urobatisjamaicensis* (Cuvier, 1816) (Elasmobranchii, Batoidea, Myliobatiformes) of the Mexican Caribbean

**DOI:** 10.3897/zookeys.1169.101968

**Published:** 2023-07-13

**Authors:** Erick Rodríguez-Ibarra, Juan Violante-González, Scott Monks

**Affiliations:** 1 Universidad Autónoma de Guerrero, Unidad Académica de Ecología Marina, Gran Vía Tropical No. 20, Fraccionamiento Las Playas, C.P. 39390, Acapulco, Guerrero, Mexico; 2 Universidad Autónoma del Estado de Hidalgo, Centro de Investigaciones Biológicas, Pachuca, Hidalgo, Mexico; 3 Universidad Autónoma del Estado de Hidalgo, Centro de Investigaciones Biológicas, Apartado Postal 1-10, C.P. 42001, Pachuca, Hidalgo, Mexico

**Keywords:** Elasmobranchs, marine fish parasites, Myliobatiformes, new species, tapeworms

## Abstract

Eight specimens of *Urobatisjamaicensis* were collected from four localities of the Yucatan Peninsula, Mexico, of which four specimens were infected with cestodes of two new species of *Acanthobothrium*. *Acanthobothriumgarciaprietoi***sp. nov.** differs from congeners by a combination of characters including the size of the scolex and bothridia, length of the cephalic peduncle, length of the axial and abaxial prongs and total length of the abaxial prongs of the hooks, size of the cirrus sac and testes in mature proglottids, and the total number of proglottids. The most prominent characteristic distinguishing *A.pulidofloresae***sp. nov.** from other species of the Atlantic Ocean/Caribbean Sea/Gulf of Mexico is the form of the scolex, which has the “clover leaf” configuration. In addition, it can be distinguished by the total length of the worm, total number of proglottids, small accessory suckers, the form of the bothridia, length of the axial and abaxial prongs and total abaxial prong length of hooks, and the number of testes. According to the current category scheme, *A.garciaprietoi***sp. nov.** and *A.pulidofloresae***sp. nov.**, belong to categories 1 and 5, respectively.

## ﻿Introduction

The Family Urotrygonidae (round rays) is represented by approximately 20 species in two genera, *Urobatis* Garman, 1913 and *Urotrygon* Gill, 1863. Family members are confined to tropical warm water continental shelves of the Western Atlantic and the Eastern Pacific coasts of the Americas, mainly in demersal inshore habitats (Del Moral-Flores et al. 2015; Last et al. 2016a, b). The yellow stingray, *Urobatisjamaicensis* (Cuvier, 1816) is the only member of the genus found in the Western Atlantic and Caribbean waters, with a latitudinal range from Florida to Venezuela (Lesniak et al. 2015). However, it also has been reported in North Carolina, south to Brazil, and in the Gulf of Mexico (Bigelow and Schroeder 1953; Robins et al. 1986; Hoese and Moore 1998; McEachran and Fechhelm 1998; McEachran and de Carvalho 2002; Spieler et al. 2013).

The genus *Acanthobothrium* Blanchard, 1848 currently is comprised of 211 species, and is the most diverse genus of Onchoproteocephalidea. Its members principally parasitize batoids and sharks (Caira and Jensen 2017). Recent descriptions include 23 species, eleven from the greater Atlantic Ocean (Franzese and Ivanov 2018; Rodríguez-Ibarra et al. 2018; Franzese and Ivanov 2020; Van Der Spuy et al. 2020, 2022), six from the Persian Gulf and Gulf of Oman in the northwest Indian Ocean (Maleki et al. 2018, 2019) and six from the Pacific Ocean (Zaragoza-Tapia et al. 2019, 2020b; Gallagher and Caira 2020). Of the 211 species, 17 species have been described from Mexican waters and three species known from other localities have been reported off México.

As part of a continuing study of the helminths of marine fish, eight specimens of *U.jamaicensis* were collected in the Yucatan Peninsula, Mexico; four of these were found to be infected with two species of *Acanthobothrium*. These two and ten other recently described species of *Acanthobothrium* were added to the checklist by Zaragoza-Tapia et al. (2020a). The current paper is a continuation of the process of documentation of the DAMA protocol (see Brooks et al. 2014).

## ﻿Materials and methods

Eight specimens of *Urobatisjamaicensis* were collected by local fishermen in the Yucatan Peninsula, Mexico, who donated the spiral intestines to the authors. The locations and number of rays from each location are as follows: two from Xcalak, Quintana Roo (18°15'58.37"N, 87°50'9.00"W) (collected in October 1998), one from Río Lagartos, Yucatán (21°35'58.05"N, 88°9'32.44"W) (February 1999), four from Isla Contoy, Quintana Roo (21°31'44.45"N, 86°48'11.53"W) (February 1999), and one from Isla Cozumel, El Paso de los Cedros, Quintana Roo (20°21'57.74"N, 87°1'32.48"W) (February 1999). Cestodes were removed from the spiral valve of the host, killed with hot water, transferred immediately to AFA (alcohol-formalin-acetic acid) for 24–48 hours, and then stored in 70% ethanol. Specimens were stained either with Mayer’s carmalum or Ehrlich’s hematoxylin following Pritchard and Kruse (1982), dehydrated in a graduated series of ethanol, cleared in methyl salicylate, and mounted in Canada balsam for viewing. Worms that were too large to be mounted on a single slide were cut into sections and mounted sequentially on separate slides. The prevalence of infection was calculated according to Bush et al. (1997) and Bautista-Hernández et al. (2015). Images were acquired using a Zeiss Axio Zoom V16 stereo microscope equipped with a Zeiss Axiocam M8c5 (5 megapixels) camera and controlled with the Zeiss Zen 2012 (blue edition) software.

Because the specimens had been mounted on slides in 1997–1998 (but not studied at that time), one specimen was dismounted from its single slide, placed in 100% Xylol for 24 hours, after which the excess Canada balsam was removed as much as possible with a fine brush in order to process it for scanning electron microscopy (SEM). This specimen was dehydrated through a graded series of ethanol, subjected to critical point drying with CO_2_ and then sputter coated with a gold‐palladium mixture, mounted on metal beads with silver paste; it was examined using a Hitachi Stereoscan Model S-2469 N at 15 kV (Laboratorio Nacional de Biodiversidad (LANABIO), Instituto de Biología, Universidad Nacional Autónoma de Mexico, Mexico City, Mexico. Illustrations were made with the aid of a drawing tube. Measurements are given in micrometers unless specified otherwise. The range is given, followed in parentheses by the mean, standard deviation, the number of measured worms, and the number of measurements taken. Hook measurements follow Euzet (1959), as modified by Monks et al. (1996), and Ghoshroy and Caira (2001). Other hook terminology follows (Caira 1985b). Designation of proglottid apolysis follows Caira et al. (1999) and Franzese and Ivanov (2018). In order to facilitate comparisons among species of *Acanthobothrium*, the categorical method proposed by Ghoshroy and Caira (2001) and updates by Fyler and Caira (2006) and Zaragoza-Tapia et al. (2020a) were used in conjunction with the current literature (Caira et al. 2012; Global Cestode Database). Validity of scientific names, authorities, and common names of rays follows Last et al. (2016b) and Froese and Pauly (2022). Specimens from three collections were examined.

In order to provide an update to the information in the table published by Zaragoza-Tapia et al. (2020a), ten species described after March 2020 and the two species from the current study are listed in Table 2 in the same format as in that original table; the geographic distribution were taken from Last et al. (2016b) and Froese and Pauly (2022). As an aid to taxonomists, these species were included in a revised version of the original table of Zaragoza-Tapia et al. (2020a) that can be downloaded (Suppl. material 1). The authorities of species mentioned in the Remarks are provided in Tables 1, 2.

**Table 1.** Species of *Acanthobothrium* different categories that were compared with *A.garciaprietoi* sp. nov. and *A.pulidofloresae* sp. nov. Note: * = Species with characters used are mostly assigned to category 5; ** = species with characters used are mostly assigned to another category, but some of them allow them to be in category 5; † = species considerate as species *inquirenda* by Caira et al. (2012).

Species	Type host	Type locality	Source
**Category 1**			
*A.fogeli* Goldstein, 1964	*Gymnuralessae* (Yokota & Carvalho, 2017) (as *Gymnuramicrura* (Bloch & Schneider, 1801))	Northeastern Gulf of Mexico, Florida	Goldstein (1964)
*A.himanturi* Brooks, 1977	*Styracuraschmardae* (Werner, 1904) (as *Himanturaschmardae*)	Caribbean Sea, La Cienaga, Magdalena, Colombia	Brooks (1977)
*A.lentiginosum* Vardo-Zalik & Campbell, 2011	*Pseudobatoslentiginosus* (Garman, 1880) (as *Rhinobatoslentiginosus*)	Gulf of Mexico	Vardo-Zalik and Campbell (2011)
*A.lineatum* Campbell, 1969	*Hypanusamericanus* (Hildebrand & Schroeder, 1928) (as *Dasyatisamericana*)	Chesapeake Bay, Virginia, USA	Campbell (1969)
*A.marplatensis* Ivanov & Campbell, 1998	*Atlantorajacastelnaui* (Miranda Ribeiro, 1907) (as *Riorajacastelnaui*)	Mar del Plata, Buenos Aires, Argentina	Ivanov and Campbell (1998)
*A.schalli* Vardo-Zalik & Campbell, 2011	*Musteluscanis* (Mitchill, 1815)	Gulf of Mexico	Vardo-Zalik and Campbell (2011)
*A.stefaniae* Franzese & Ivanov, 2018	*Discopygetschudii* Heckel, 1846	Coastal waters off Mar Chiquita City, Buenos Aires Province	Franzese and Ivanov (2018)
*A.ulmeri* Vardo-Zalik & Campbell, 2011	*Rostrorajatexana* Chandler, 1921 (as *Rajatexana*)	Gulf of Mexico	Vardo-Zalik and Campbell (2011)
*A.westi* Vardo-Zalik & Campbell, 2011	*R.texana*	Gulf of Mexico	Vardo-Zalik and Campbell (2011)
**Category 2**			
*A.tasajerasi* Brooks, 1977	*S.schmardae*	Caribbean Sea, La Cienaga, Magdalena, Colombia	Brooks (1977)
*A.cartagenensis* Brooks & Mayes, 1980	*Urobatisjamaicensis* (Cuvier, 1816)	Cartagena, Colombia	Brooks and Mayes (1980)
*A.urotrygoni* Brooks & Mayes, 1980	*Urotrygonvenezuelae* (Schultz, 1949)	Cartagena, Colombia	Brooks and Mayes (1980)
**Category 1 and 2**			
*A.carolinae* Franzese & Ivanov, 2020	*Bathyrajamagellanica* (Philippi, 1902)	Coastal waters off Puerto San Julián, Santa Cruz Province	Franzese and Ivanov (2020)
*A.domingae* Franzese & Ivanov, 2020	*Dipturusbrevicaudatus* (Marini, 1933)	Coastal waters off Santa Teresita, Buenos Aires Province	Franzese and Ivanov (2020)
**Category 3**			
*A.majumdari* Pramanik & Manna, 2010†	*Carchariaswalbeehmii* (Bleeker, 1856)	Digha coast, India	Pramanik and Manna (2010)
*A.robertsoni* Campbell & Beveridge, 2002	*Trygonorrhinafasciata* Müller & Henle, 1841	Middleton, South Australia	Campbell and Beveridge (2002)
*A.zugeinensis* Yang & Lin, 1994†	*T.zugei* (as *Dasyatiszugei*)	Xiamen, South Fujian, China	Yang and Lin (1994)
**Category 4**			
*A.adlardi* Campbell & Beveridge, 2002	*Pristiophoruscirratus* (Latham, 1794)	Port Stanvac, South Australia	Campbell and Beveridge (2002)
*A.dasybati* Yamaguti, 1934 (Yamaguti, 1952)	*Hemitrygonakajei* (Müller & Henle, 1841) (as *Dasyatisakajei*)	Tarumi, Kobe, Japan	Yamaguti (1934)
*A.cestraciontis* (Yamaguti, 1934)	*Heterodontusjaponicus* (Miklouho-Maclay & Macleay, 1884) (as *Cestracionjaponicus*)	Pacific Ocean, Japan	Yamaguti (1934)
*A.cribbi* Campbell & Beveridge, 2002	*Gymnuraaustralis* (Ramsay & Ogilby, 1886)	Gulf of Carpentaria, Northern Territory, Australia	Campbell and Beveridge (2002)
*A.micracantha* Yamaguti, 1952	*H.akajei*	Nagasaki, East China Sea, Japan	Yamaguti (1952)
*A.grandiceps* Yamaguti, 1952	*Telatrygonzugei* (Müller & Henle, 1841) (as *Trygonzugei*)	East China Sea, Japan	Yamaguti (1952)
*A.ijimai* Yoshida, 1917	*H.akajei*	Tokyo, Japan	Yoshida (1917), Williams (1969), Yang et al. (2016)
*A.karachiense* Bilqees, 1980	*Mustelusmanazo* Bleeker, 1855 (as *Cyniasmanazo*)	Karachi Coast, Pakistan	Bilqees (1980)
*A.macrocephalum* Wang & Yang, 2001	*H.akajei*	Xiamen, Fujiari, China	Wang and Yang (2001)
*A.pingtanensis* Wang, 1984	*Neotrygonkuhlii* (Müller & Henle, 1841) (as *Dasyatiskuhlii*)	Fujian Province, China	Wang (1984)
**Category 5**			
*A.amazonensis* Mayes, Brooks & Thorson, 1978	*Potamotrygoncircularis* (German, 1913)	Itacuari River, Brazil	Mayes et al. (1978)
*A.angelae* Campbell & Beveridge, 2002	*Hypnosmonopterygius* (Shaw, 1795)	Yarraville Shoals, South Australia	Campbell and Beveridge (2002)
*A.confusum* Baer & Euzet, 1962	*N.kuhlii*	Indian Ocean, Sri Lanka	Baer and Euzet (1962)
*A.edmondsi* Campbell & Beveridge, 2002	*Parascylliumferrugineum* (McCulloch, 1911)	Port Stanvac, South Australia	Campbell and Beveridge (2002)
*A.franus* Marques, Centritto & Stewart, 1997*	*Narcineentemedor* (Jordan & Starks, 1895)	Cuajiniquil Beach, Gulf of Santa Helena, Guanacaste, Costa Rica	Marques et al. (1997b)
*A.giganticum* Sanaka, Lakshmi & Hanumantharao, 1993	*G.micrura*	Waltair coast, India	Sanaka et al. (1993)
*A.goldsteini* Appy & Dailey, 1973*	*Platyrhinoidistriseriata* (Jordan & Gilbert, 1880)	Seal Beach, California, USA	Appy and Dailey (1973)
*A.hispidum* Riser, 1955	*Tetronarcecalifornica* (Ayres, 1855)	Monterey Bay, California, USA	Riser (1955)
*A.inbiorium* Marques, Centritto & Stewart, 1997	*N.entemedor*	Cuajiniquil Beach, Gulf of Santa Helena, Guanacaste, Costa Rica	Marques et al. (1997b)
*A.indicum* (Subhapradha, 1955)	*Narcinebrasiliensis* (Olfers, 1831) (as *Narcinebraunii*)	Madras Coast, India	Subhapradha (1955)
*A.katherineae* Gallagher & Caira, 2020	*Squaliolusaliae* (Teng, 1959)	Taiwan Strait, landed at Donggang, Pingtung Province, Taiwan	Gallagher and Caira (2020)
*A.lintoni* Goldstein, Henson & Schlicht, 1968**	*N.brasiliensis*	Gulf of Mexico, Texas, USA	Goldstein et al. (1969)
*A.manteri* Hassan, 1983	*Pastinachussephen* (Forsskål, 1775) (as *Dasyatissephen*)	Mediterranean Sea, Egypt	Hassan (1983)
*A.maryanskii* Caira & Burge, 2001	*Diplobatisommata* (Jordan & Gilbert, 1890)	Loreto, Gulf of California, Mexico	Caira and Burge (2001)
*A.paulum* Linton, 1890**	*Bathytoshiacentroura* (Mitchill, 1815) (as *Trygoncentrura* [sic])	Woods Hole, Massachusetts, USA	Linton (1890), Vardo- Zalik and Campbell (2011)
*A.psammobati* Carvajal & Goldstein, 1969	*Psammobatisscobina* (Philippi, 1857)	South Pacific Ocean, between Papudo and Talcahuano, Chile	Carvajal-G. and Goldstein (1969)
*A.quinonesi* Mayes, Brooks & Thorson, 1978	*Potamotrygonmagdalenae* (Duméril, 1865)	Magdalena River, Cienaga Jobo, vicinity of San Cristobal, Bolivar, Colombia	Mayes et al. (1978)
*A.rajaebatis* (Rudolphi, 1810) Euzet, 1959	*Dipturusoxyrinchus* (Linnaeus, 1758) (as *Rajabati*)	Mediterranean Sea	Rudolphi (1810)
*A.regoi* Brooks, Mayes & Thorson, 1981	*Potamotrygonhystrix* (Müller & Henle, 1841)	Orinoco River Delta, Orinoco River near Los Castillos, Venezuela	Brooks et al. (1981)
*A.rhinobati* Alexander, 1953**	*Pseudobatosproductus* (Ayres, 1854) (as *Rhinobatosproductus*)	Santa Monica Harbor, California, USA	Alexander (1953)

**Table 2.** Species of *Acanthobothrium* reported from the different species of elasmobranchs of the world (update of Zaragoza-Tapia, 2020a). Abbreviations: **Gd** = Geographical distribution; **Ht** = Holotype; **Nt** = Neotype; **Pt** = Paratype; **Va** = Voucher; **Loc** = Locality; **Sou** = Source; **Cat** = Category designation (modified from Zaragoza-Tapia 2020a).

Species	Ht	Nt, Pt or Va	Host	Gd	Loc	Sou	Cat
*A.carolinae* Franzese & Ivanov, 2020	MACN-Pa 716	MACN-Pa 717/1-4, 718/1-3, 719/1-2; IPCAS C-838; LRP 10179-10184	*Bathyrajamagellanica* (Philippi, 1902)	WSA, ESP	Coastal waters off Puerto San Julián, Santa Cruz Province, Argentina	Franzese and Ivanov (2020)	1 and 2
*A.carolinae*	NR	NR	*Bathyrajamagellanica*	WSA, ESP	Coastal waters off Río Grande, Tierra del Fuego Province, off Banco Burdwood, Argentina	Franzese and Ivanov (2020)	–
*A.domingae* Franzese & Ivanov, 2020	MACN-Pa 720	MACN-Pa 721/1-3, 722/1-9, 723; IPCAS C-839; LRP 10185-10195	*Dipturusbrevicaudatus* (Marini, 1933)	WSA	Coastal waters off Santa Teresita, Buenos Aires Province, Argentina	Franzese and Ivanov (2020)	1 and 2
*A.domingae*	NR	NR	*Dipturusbrevicaudatus*	WSA	Coastal waters off Río Grande, Tierra del Fuego Province, off Banco Burdwood, Argentina	Franzese and Ivanov (2020)	–
*A.microhabentes* Van Der Spuy, Smit & Schaeffner, 2020	NMB P-604	NMB P-605-P-606; IPCAS C-848; MHNG PLAT-137324, 137334	*Rajastraeleni* (Poll, 1951)	ESA, ECA	South-eastern Atlantic Ocean off De Kelders, South Africa	Van Der Spuy et al. (2020)	2
*A.microtenuis* Van Der Spuy, Smit & Schaeffner, 2020	NMB P-607	NMB P-608-P-609; IPCAS C-849; MHNG PLAT-137335, 137340	*Rajastraeleni*	ESA, ECA	South-eastern Atlantic Ocean off De Kelders, South Africa	Van Der Spuy et al. (2020)	2
*A.crassus* Van Der Spuy, Smit & Schaeffner, 2020	NMB P-610	NMB P-611; IPCAS C-850; MHNG PLAT-137341	*Rajastraeleni*	ESA, ECA	South-eastern Atlantic Ocean off De Kelders, South Africa	Van Der Spuy et al. (2020)	2
*A.dolichocollum* Van Der Spuy, Smit & Schaeffner, 2020	NMB P-612	NMB P-613; IPCAS C-851; MHNG PLAT-137342, 137343	*Rajastraeleni*	ESA, ECA	South-eastern Atlantic Ocean off Hermanus, South Africa	Van Der Spuy et al. (2020)	2
*A.umbungus* Van Der Spuy, Smit & Schaeffner, 2022	NR	NR	*Rostrorajaalba* (Lacépède, 1803)	ENA, MED, ECA, ESA, WIO	Danger Point, Gansbaai, South Africa	Van Der Spuy et al. (2022)	2
*A.usengozinius* Van Der Spuy, Smit & Schaeffner, 2022	NR	NR	*Rostrorajaalba*	ENA, MED, ECA, ESA, WIO	Danger Point, Gansbaai, South Africa	Van Der Spuy et al. (2022)	2
*A.ulondolozus* Van Der Spuy, Smit & Schaeffner, 2022	NR	NR	*Rostrorajaalba*	ENA, MED, ECA, ESA, WIO	Danger Point, Gansbaai, South Africa	Van Der Spuy et al. (2022)	2
*A.katherineae* Gallagher & Caira, 2020	NMNS 8249-001	LRP 10260, 10015, 10016; USNM 1618754, 1618752, 1618753	*Squaliolusaliae* (Teng, 1959)	EIO, NIO, WCP, WNP, WSP	Taiwan Strait, landed at Donggang, Pingtung Province, Taiwan	Gallagher and Caira (2020)	5
*A.garciaprietoi* sp. nov.	CNHE 11881	CNHE 11882, HWML-216977, CHE-P00146	*Urobatisjamaicensis* (Cuvier, 1816)	WCA, WNA	Isla Contoy, Playa Ixmapoit, Quintana Roo, Mexico	This studio	1
*A.pulidofloresae* sp. nov.	CNHE 11880	CHE P00145	*Urobatisjamaicensis*	WCA, WNA	Isla Cozumel, El Paso de los Cedros, Quintana Roo, Mexico	This studio	5
*A.barusi* Pramanik & Manna, 2010	Department of Zoology, University of Calcutta #004	Department of Zoology, University of Calcutta #003	*Aetobatusnarinari*	NIO	Digha coast, West Bengal, India	Pramanik and Manna (2010)	3 (4)
*A.majumdari* Pramanik & Manna, 2010	Department of Zoology, University of Calcutta #0013	Department of Zoology, University of Calcutta #003	*Carchariaswalbeehmi* Bleeker, 1856	NIO	Digha coast, India	Pramanik and Manna (2010)	3(4)
*A.paramanandai* Pramanik & Manna, 2010	Department of zoology, University of Calcutta #002	Department of zoology, University of Calcutta #003	*Carcharhinushemiodon* (Valenciennes, 1839)	NIO	Digha coast, West Bengal, India	Pramanik and Manna (2010)	3(4)
*A.tsingtaoensis* Tseng, 1933	NR	NR	*Hemitrygonakajei*	WNP	China	Tseng (1933)	–
*A.zugeinensis* Yang & Lin, 1994	NR	NR	*Telatrygonzugei*	WNP	Xiamen, South Fujian, China	Yang and Lin (1994)	3

**Legend: Collections**: Colección de Helmintos, Centro de Investigaciones Biológicas, Universidad Autónoma del Estado de Hidalgo, Pachuca, México (**CHE**); Colección Nacional de Helmintos del Instituto de Biología, Universidad Nacional Autónoma de México, México (**CNHE**); University of Nebraska State Museum, Harold W. Manter Laboratory, Division of Parasitology, Lincoln, Nebraska, United States (**HWML**); Institute of Parasitology, Academy of Sciences of the Czech Republic, České Budějovice, Czech Republic (**IPCAS**); Lawrence R. Penner Parasitology Collection, Helminthological Collection, University of Connecticut, Storrs, Connecticut, United States (**LRP**); Museo Argentino de Ciencias Naturales, Colección Parasitológica, Buenos Aires, Argentina (**MACN-Pa**); Natural History Museum, Geneva, Switzerland (**MHNG-PLAT**); National Museum, Bloemfontein, South Africa (**NMB**); National Museum of Natural History, Smithsonian Institution, Washington, D.C., United States (**USNM**). “**NR**” was used for data that are not reported in the original source. **Geography**: Eastern Central Atlantic (**ECA)**; Eastern Indian Ocean (**EIO**); Eastern North Atlantic (**ENA**); Eastern South Atlantic (**ESA**); Eastern South Pacific (**ESP**); Mediterranean Sea (**MED**); Northern Indian Ocean (**NIO**); Western Central Atlantic (**WCA**); Western Central Pacific (**WCP**); Western Indian Ocean (**WIO**); Western North Atlantic (**WNA**); Western North Pacific (**WNP**); Western South Atlantic (**WSA**); Western South Pacific (**WSP**).

### ﻿Abbreviations

**CNHE** Colección Nacional de Helmintos del Instituto de Biología, Universidad Nacional Autónoma de México, México;

**HWML** University of Nebraska State Museum, Harold W. Manter Laboratory, Division of Parasitology, Lincoln, Nebraska, USA;

**CHE** Colección de Helmintos, Centro de Investigaciones Biológicas, Universidad Autónoma del Estado de Hidalgo, Pachuca, México.

Type material was deposited in **CNHE** (holotype and paratypes), **HWML** (paratypes), and **CHE** (paratypes).

## ﻿Systematic accounts

### ﻿Order Onchoproteocephalidea Caira, Jensen, Waeschenbach, Olson & Littlewood, 2014

**Family Onchobothriidae Braun, 1900**

#### ﻿Genus Acanthobothrium

Taxon classificationAnimaliaOnchoproteocephalideaOnchobothriidae

Blanchard, 1848

F509E24A-6ED3-5DAB-90F3-34347BDADF6E

##### Type species.

*Acanthobothriumcoronatum* (Rudolphi, 1819) Blanchard, 1848

#### ﻿ Acanthobothrium pulidofloresae sp. nov.

Taxon classificationAnimaliaOnchoproteocephalideaOnchobothriidae

AED3FCA9-C2E7-58EF-8396-9AE2DC1FF485

https://zoobank.org/8278E3A7-55EF-46EF-BBD3-CAD68BA8041B

Figs 1 , 2

##### Type material.

***Holotype*** (CNHE–11880), 1 ***paratype*** (CHE-P00145).

##### Type host.

*Urobatisjamaicensis* (Cuvier, 1816) (Elasmobranchii: Myliobatiformes: Urotrygonidae).

##### Type locality.

Isla Cozumel, El Paso de los Cedros, (20°21'57.74"N, 87°1'32.48"W), Quintana Roo, Mexico.

##### Site of infection.

Spiral valve.

##### Prevalence of infection.

14.28%

##### Etymology.

The species is named in honor of Dra. Griselda Pulido-Flores (Universidad Autónoma del Estado de Hidalgo, Centro de Investigaciones Biológicas, Hidalgo, Mexico) for her contributions to the academic training of the first author, and to knowledge of helminths, as well as for her friendship.

##### Diagnosis.

*Acanthobothriumpulidofloresae* sp. nov. is a Category 5 species. Body relatively long (41 mm long); scolex in a “clover leaf” configuration; apical suckers small, 12–13 (12.6 ± 2; 1; 3) long by 30–32 (31.33 ± 3; 1; 3) wide; strobila of 152–164 proglottids; short hooks, in claw configuration, 47–60 testes per proglottid; ovary symmetrical.

##### Description.

**Figure 1.**
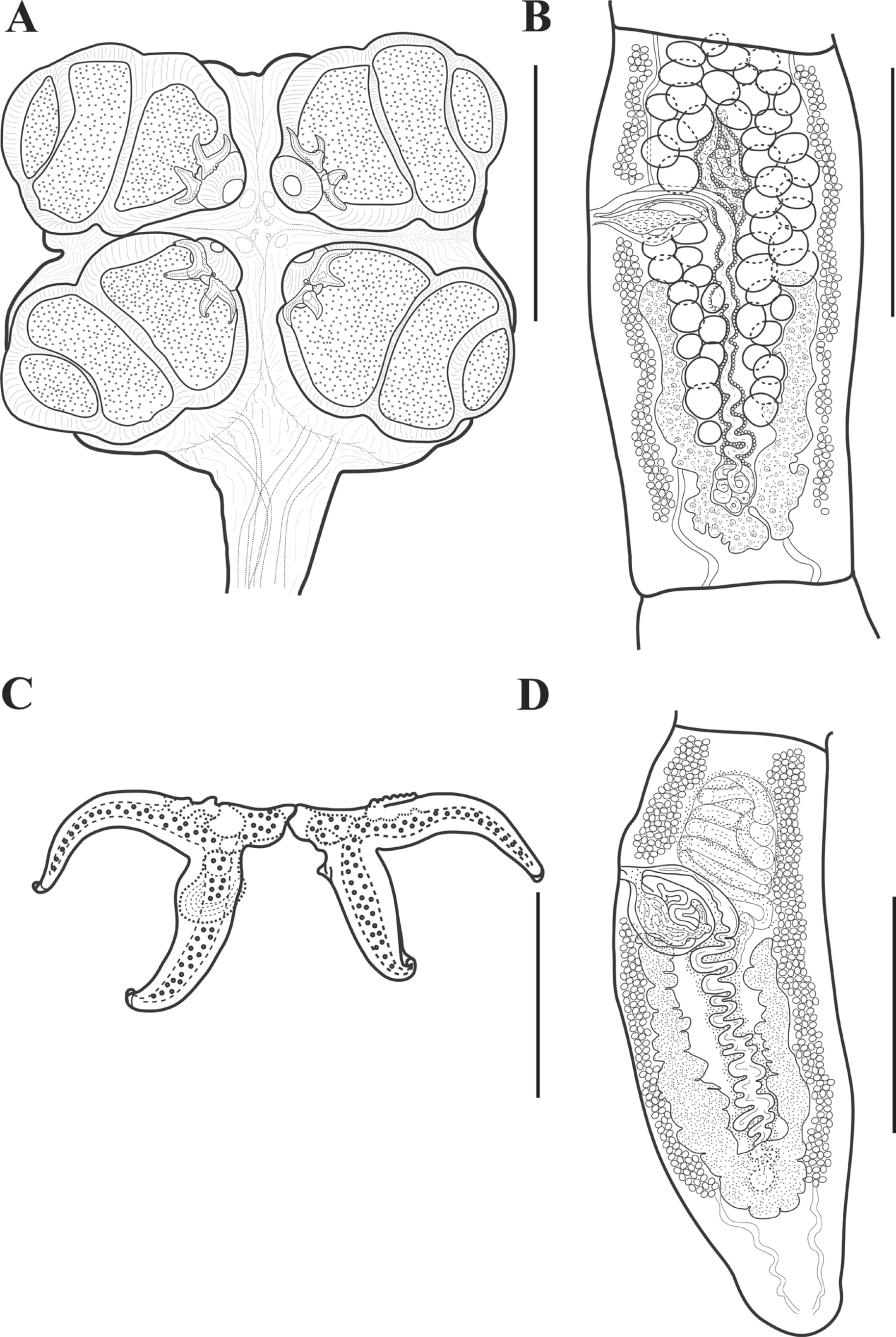
*Acanthobothriumpulidofloresae* sp. nov. **A, C, D** holotype (CNHE–11880) **B** paratype (CHE-P00145) **A** scolex **B** mature proglottid **C** pair of hooks **D** terminal mature proglottid. Scale bars: 75 μm (**C**); 400 μm (**B, D**); 500 μm (**A**).

**Figure 2.**
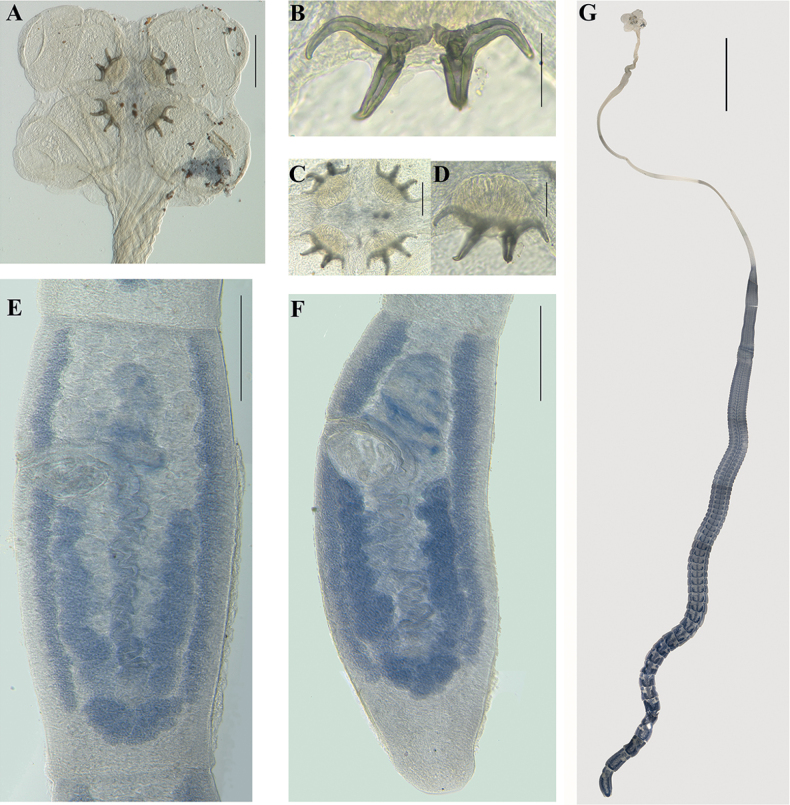
*Acanthobothriumpulidofloresae* sp. nov. **A–D, G** holotype (CNHE–11880) **E, F** paratype (CHE-P00145) **A** scolex **B** pair of hooks **C** apical suckers with pads muscular **D** apical sucker **E** mature proglottid **F** terminal mature proglottid **G** complete tapeworm. Scale bars: 50 μm (**B, D**); 100 μm (**C**); 200 μm (**A, E**, **F**); 3 mm (**G**).

(Based upon measurements of one complete worm and one partial worm mounted on slides). Entire worm 41 mm long, greatest width at scolex (Figs 1, 2), euapolytic; 152–164 (158; 2) proglottids per worm (Figs 1, 2). Scolex consisting of scolex proper and cephalic peduncle; petaloid scolex proper, 288 long by 413 wide, maximum width of scolex at level of posterior end of posterior loculus (because of petaloid shape); floor of loculi surfaces and other surfaces of scolex without obvious microtriches (Figs 1A, 2A). Bothridia attached to velum only from middle loculi to anterior end; velum flexible, when relaxed allows bothridia be in a position perpendicular to the rest of the scolex (i.e., petaloid scolex). Bothridia 475–515 (487 ± 32; 1; 4) long by 350–395 (378 ± 32; 1; 4) wide; each with three loculi separated by transverse septa, anterior region specialized, in form of muscular pad. Muscular pad 60–65 (62.5 ± 6; 1; 4) long, 114–126 (118 ± 8; 1; 4) wide, with apical sucker not well defined, 12–13 (12.6 ± 2; 1; 3) long by 30–32 (31.33 ± 3; 1; 3) wide. Each pad with one pair of bipronged hooks at posterior margin. Anterior loculus of bothridia 160–175 (170 ± 7; 1; 4) long, middle loculus 115–130 (123 ± 6; 1; 4) long, posterior loculi 75–100 (81 ± 13; 1; 4) long; ratio of lengths of loculus (anterior:middle:posterior) 1:0.7:0.5. Velum present between medial margins of bothridia at posterior margin of middle loculus (Figs 1A, 2A).

Hooks with axial and abaxial prongs, hollow, with tubercle on proximal surface of axial prong; internal channels of axial and abaxial prongs continuous, smooth; base and anterior part of each hook embedded in muscular pad; handle short and thin, prongs short with tips turned inwards; abaxial prongs longer than axial prongs. Bases (handles) of medial and lateral hooks articulated to each other; lateral hooks relatively longer than internal hooks. Lateral hook measurements: A 36–42 (39 ± 3; 1; 4), B 48–60 (51 ± 7; 1; 4), C 57–68 (62.4 ± 8; 1; 4), D 76–87 (81 ± 7; 1; 4). Medial hook measurements: A’ 38–42 (40.8 ± 3; 1; 4), B’ 39–57 (36 ± 6; 1; 3), C’ 48–59 (52 ± 6; 1; 4), D’ 73–80 (76 ± 4; 1; 3) (Figs 1C, 2B).

Cephalic peduncle 14.6 mm long, 220–317 (268 ± 69, n = 2) wide at mid-level; presence and type of microtriches not confirmed. Proglottids craspedote. Immature proglottids 230–500 (369 ± 81; 2; 8) long by 537–866 (708 ± 157; 2; 8) wide; mature proglottids, 537–1000 (753 ± 181; 2; 7) long by 476–769 (627 ± 137; 2; 7) wide; terminal mature proglottids 915–1793 (1196 ± 344; 2; 5) long by 415–633 (525 ± 105; 2; 5) wide. Gravid proglottids not observed. Genital pores lateral, irregularly alternating, 23–37% (29 ± 5; 2; 6) of total length of proglottid from anterior end in mature proglottids; in terminal mature proglottids, 22–43% (29 ± 9; 2; 5); genital atrium shallow. Testes oval in dorsoventral view, arranged irregularly anterior to ovarian lobes, two layers deep, 48–78 (64 ± 8; 2; 24) long by 24–59 (44 ± 8; 1; 24) wide (Figs 1B, 2E). Testes 47–60 (52 ± 3; 2; 22) in total number, 24–33 (28 ± 2; 2; 22) aporal, 9–15 (11 ± 2; 2; 22) preporal, 10–16 (13 ± 2; 2; 22) postporal; no testes posterior to ovarian isthmus. Cirrus sac pyriform, curved posteriorly, 144–192 (166 ± 18; 2; 6) long by 68–96 (86 ± 13; 2; 6) wide in mature proglottid, efferent vessel is posterior in reference to the cirrus sac. Cirrus with spinitriches, eversible, globose. Vagina anterior to cirrus sac, vaginal wall glandular. Vaginal canal forms strongly marked loops, which descend slightly aporal to midline of proglottid; glandular cells extend along canal wall. Ovary with approximately symmetrical arms, follicular; poral lobe 210–500 (349 ± 99; 2; 12) long in mature proglottids, aporal lobe 218–510 (350 ± 95; 2; 12) long, extending to the posterior margin of cirrus sac in terminal proglottids (Figs 1B, D, 2E, F). Shape of ovary in frontal view changes as proglottids mature: almost U-shaped in immature proglottids, inverted A-shaped in mature proglottids and inverted A– or V-shaped inverted in terminal proglottids; posterior arms of ovary are overlapping but appear joined. In mature and terminal proglottids, isthmus is located at middle of uterus. Mehlis’ gland posterior to isthmus; seminal receptacle not seen. Vitellarium follicular, consisting of two lateral bands; each band consisting of two columns of relatively large elongate oval follicles extending from near anterior margin of proglottid to near posterior margin, interrupted by vagina and cirrus sac; follicles 770–985 (842± 82; 2; 7) long, 60–100 (76 ± 19; 2; 7) wide. Uterus thin-walled, sacciform, extends from near anterior part of proglottid to near oӧtype. Excretory ducts lateral. Eggs not seen.

##### Remarks.

For *U.jamaicensis*, this is the second species of *Acanthobothrium* that has been described. *Acanthobothriumcartagenensis* was designated to Category 1, recently reassigned by Zaragoza-Tapia et al. (2020a). *Acanthobothriumpulidofloresae* sp. nov. belongs to Category 5 (sensu Ghoshroy and Caira 2001). Category 5 species have a total length > 15 mm (the new species is 41 mm long); number of proglottids > 50 proglottids (the new species has 152–164 proglottids); the number of testes per proglottids ≤ 80 (the new species has 47–60 testes per proglottid); and a symmetrical ovary (which the new species has).

*Acanthobothriumpulidofloresae* sp. nov. is easily distinguished from the other species of Category 5 because none of them has a scolex configuration called “clover leaf” (Fyler and Caira 2010; Yoshida 1917); *A.pulidofloresae* sp. nov. also is distinguished from *A.lintoni* (2.5–22.62 mm) and *A.paulum* (4.5–20 mm) because it is longer (41 mm) and has a greater number of proglottids (*A.pulidofloresae* sp. nov. has 152–164 long vs. *A.lintoni* = 5–60 and *A.paulum* = 16–20). The new species has smaller accessory suckers (12–13 long by 30–32 wide vs. 42–95 and 60–70, respectively); the bothridia are short (425–470 long vs. 389–720, and 500–800, respectively); much shorter hooks (total abaxial length 78–94 vs. 108–230, and 140–200, respectively). The new species has a greater number of testes (47–60 vs. 30–46, and 40–45, respectively) (Table 1).

*Acanthobothriumpulidofloresae* sp. nov. differs from *A.amazonensis* and *A.quinonesi* because it is relatively longer; however, *A.regoi* is approximately the same size (41 mm vs. *A.amazonensis* = 35, *A.quinonesi* = 25, and *A.regoi* = 45). These species also differ in the number of proglottids (152–164 vs. 75–100, 55–75, 87–120, respectively). The new species has a smaller accessory sucker (12–13 long by 30–32 wide vs. 85–107, 53–66, 61–102 in diameter, respectively). The hooks are shorter (total abaxial length 78–94 vs. 145–184, 100–142, 122–163, respectively) and the number of testes is less (47–60 vs. 50–72 in *A.amazonensis*, 43–62 in *A.quinonesi*, 47–70). The new species differs also from *A.manteri* because that species is slightly longer (the new species is 41 mm long vs. *A.manteri* 36–65 mm long. However, it is smaller than *A.rajaebatis* (41 mm long vs. 50–60 mm); has a smaller accessory sucker (12–13 long by 30–32 wide vs. 198 and 312 in diameter), shorter hooks (total abaxial length 78–94 vs. 175 and 305 and fewer testes per proglottids (47–60 vs. 55–74 and 58–85). Finally, it differs in the number of proglottids but there is some overlap in range (152–164 for *A.pulidofloresae* sp. nov. vs. 120–170 for *A.manteri*, and 80–120 for *A.rajaebatis*).

The new species is different from *A.franus* (16–40 long, 68–141 proglottids) and *A.psammobati* (34 long, 90 proglottids) by being longer and having a greater number of proglottids. *Acanthobothriuminbiorium* (28–82 long, 156–223 number of proglottids) is longer and has a greater number of proglottids than *A.pulidofloresae* sp. nov. In addition, *A.franus*, *A.psammobati* and *A.inbiorium* differ by presenting accessory sucker larger in diameter (*A.pulidofloresae* sp. nov. 12–13 vs. 60–159, 50, 20–75, respectively), bothridium longer (*A.pulidofloresae* sp. nov. 425–470 vs. 627–1408, 605, 480–680, respectively). *Acanthobothriumpulidofloresae* sp. nov. and *A.psammobati* have hooks that are relatively equal in length (*A.pulidofloresae* sp. nov., total abaxial length = 78–94 vs. *A.psammobati* = 91) and the hooks of the new species are shorter in length than those of *A.franus* (354–465) and *A.inbiorium* (95–120). *Acanthobothriumpulidofloresae* sp. nov. differs in the total number of testes (47–60) from *A.franus* (24–59), and *A.psammobati* (77), although there is some overlap with *A.ibiorium* (44–73).

Finally, *A.angelae*, *A.confusum*, *A.edmondsi*, *A.giganticum*, *A.hispidium*, and *A.xiamenensis* are worms with range in lengths of 50–240 mm vs. the 41 mm length of *A.pulidofloresae* sp. nov. *Acanthobothriumangelae*, *A.confusum*, *A.giganticum*, *A.hispidium*, *A.maryanskii*, *A.xiamenensis* have 250–500 proglottids, all more than *A.pulidofloresae* sp. nov. (152–164 proglottids), and *A.edmondsi* (47–86), *A.goldsteini* (60), and *A.rhinobati* (50) have a lesser number of proglottids than the new species. *Acanthobothriumindicum* has approximately the same number of proglottids as *A.pulidofloresae* sp. nov. (152–164 proglottids) but it is smaller in overall length (41 mm vs. 25 mm of *A.indicum*), and the new species has less testes per proglottid than *A.indicum* (47–60 vs. 70 of *A.indicum*). *Acanthobothriumkatherineae* is shorter than the new species (21 vs. 41 mm of *A.pulidofloresae* sp. nov.), the anterior loculus is longer that of *A.pulidofloresae* (336–417 vs. 160–175, respectively), and it has a greater number of testes (*A.katherineae* = 55–69 vs. *A.pulidofloresae* sp. nov. = 47–60). *Acanthobothriumangelae*, *A.confusum*, *A.edmondsi*, *A.giganticum*, *A.goldsteini*, *A.maryanskii*, and *A.xiamenensis* have an accessory sucker greater in diameter than that of *A.pulidofloresae*.

The following species of *Acanthobothrium* are described as having a clover-leaf scolex configuration: Category 3, *A.robertsoni*; Category 4, *A.adlardi*, *A.cestraciontis*, *A.dasybati*, *A.grandiceps*, *A.karachiense*, *A.ijimai*, *A.macrocephalum* and *A.pingtanensis*; *A.mujibi* has not been assigned to a category because of the lack of data in the description. *Acanthobothriumcribbi* is illustrated as having a scolex with bothridia that extend almost to have a petaloid scolex; however, the species is not described as having a clover-leaf scolex configuration (Campbell and Beveridge 2002) (Table 1).

*Acanthobothriumpulidofloresae* sp. nov. differs from the ten species with a petaloid scolex in the following characteristics: *A.robertsoni* is longer than the new species (83 mm vs. 41 mm of *A.pulidofloresae* sp. nov.), has a greater number of proglottids (395 vs. 152–164), the scolex is larger (3000–3250 vs. 288), the accessory suckers are larger (104–152 vs. 12–13 long by 30–32 wide), the bothridia and loculi are longer (1840–2220 vs. 425–470), the hooks are longer in total abaxial length (133–208 vs. 84–90), and the number of testes is greater (116–163 vs. 47–60). *Acanthobothriumadlardi* has a greater number of proglottids (235 vs. 152–164), the scolex is larger (800–1500 vs. 288), the accessory suckers are reported as being absent, the bothridia (and loculi) are longer (1100–1500 vs. 425–470), the hooks are longer in total abaxial length (148–176 vs. 84–90), and the number of testes is greater (83–103 vs. 47–60). *Acanthobothriumcestraciontis* differs from the new species by being longer (282 mm vs. 41), the accessory suckers are reported as being absent, the hooks are longer in total abaxial length (270–290 vs. 84–90), and the number of testes is greater (120 vs. 47–60). *Acanthobothriumdasybati* differs by being longer (52 mm), less proglottids (100), the accessory suckers are reported as being absent, the hooks are longer in total abaxial length (130 vs. 84–90), and the number of testes is greater (90–100 vs. 47–60). *Acanthobothriumgrandiceps* differs from the new species by being shorter (14–20 mm vs. 41 mm), having a greater number of proglottids (950 vs. 152–164), the accessory suckers are larger (200–210 vs. 12–13 long by 30–32 wide), the bothridia (and loculi) are longer (2200–2300 vs. 427–460), the hooks are longer in total abaxial length (165–180 vs. 84–90), and the number of testes is greater (110–130 vs. 47–60). *Acanthobothriumkarachiense* is different from the new species by having a greater number of proglottids (278–293 vs. 152–164, the scolex is larger (1750–1790 vs. 288), the bothridia (and loculi) are longer (1290–1450 vs. 427–460), it has smaller hooks in total abaxial length (62–68 vs. 84–90), and the number of testes is greater (74–98 vs. 47–60). *Acanthobothriumijimai* differs from the new species by having larger accessory suckers (100 vs. 12–13 long by 30–32 wide), the bothridia are longer (1600–1700 vs. 425–470), the hooks are longer in total abaxial length (90–110 vs. 84–90), and the number of testes is greater (100–130 vs. 47–60). *Acanthobothriummacrocephalum* differs from the new species by being longer (82–287 mm vs. 41), having a greater number of proglottids (209–1000 vs. 152–164), the scolex is larger (3000–3250 vs. 288), the accessory suckers are larger (146–308 vs. 12–13 long by 30–32 wide), the bothridia (and loculi) are longer (1230–2471 vs. 425–470), the hooks are longer in the total abaxial length (96–142 vs. 84–90), and the number of testes is greater (92–145 vs. 47–60). *Acanthobothriumpingtanensis* differs from the new species by being longer (150–160 mm vs. 41 mm), the scolex is larger (3200–3680 vs. 288), the accessory sucker is larger (320–340 in diameter vs. 12–13 long by 30–32 wide), and the total number of testes is greater (92–106 vs. 47–60). *Acanthobothriummujibi* differs from the new species by having a larger scolex (750–800 vs. 288 of *A.pulidofloresae* sp. nov.), bothridia that are longer (930 vs. 427–460), smaller hooks in total abaxial length (58–76 vs. 84–90), and the number of testes is less (36–41 vs. 47–60). Finally, *A.cribbi* is different from *A.pulidofloresae* sp. nov. by being somewhat larger (50 mm vs. 41 mm), having a greater number of proglottids (200–300 vs. 152–164), larger accessory suckers (80–152 in diameter vs. 12–13 long by 30–32 wide), and a greater total number of testes (72–96 vs. 47–60).

Three other species cataloged as *species inquirendae* by Caira et al. (2012) are illustrated as having a clover-leaf scolex configuration: *A.majumdari*, *A.tsingtaoensis*, and *A.zugeinensis*. *Acanthobothriummajumdari* and *A.zugeinensis* can be considered to be in Category 3 based on the symmetry of the ovarian lobes, i.e., for *A majumdari*, which which was reported as “each lobe length 0.1 (0.07–0.11)” (Pramanik and Manna 2010), and *A.zugeinensis* described as “ovary lobed 902–985 µm for 213–236 µm” (Yang and Lin 1994). It is not possible to determine a possible category for *A.tsingtaoensis*. *Acanthobothriummajumdari* differs from the new species by being longer (60–67 mm vs. 41 mm), having a very wide scolex (4400–5100 µm; i.e.,“4.8 (4.4–5.1) mm”) (Pramanik and Manna 2010) vs. 288 long by 413 µm wide for the new species; the bothridia are longer (2000 µm vs. 425–470 µm), the hooks are smaller in *A.majumdari* (total hook length 70 µm vs. 78–94 µm), the handle is longer (51–55 µm vs. 36–42), the number of testes per proglottid in *A.majumdari* is greater (145–155 vs. 47–60), and *A.majumdari* is apolytic.

*Acanthobothriumpulidofloresae* sp. nov. differs from *A.zugeinensis* by being shorter (41 mm vs. 140–160 mm), having a shorter scolex (288 long by 413 µm wide vs. 2601 by 2506 µm, see Yang and Lin 1994), the bothridia are shorter (425–470 µm vs. 664–688 µm), the hooks are longer (78–94 µm total hook length vs. 68–75 µm), the length of the handle is shorter (36–42 vs. 51–55 µm). The new species differs from *A.majumdari* in the number of testes per proglottid (47–60 vs. 106–132) and it can be distinguished from *A.majumdari* because the latter species is acraspedote.

#### ﻿ Acanthobothrium garciaprietoi sp. nov.

Taxon classificationAnimaliaOnchoproteocephalideaOnchobothriidae

AD2ECDD0-9C78-5288-88ED-3AFCA159E3F8

https://zoobank.org/6CE1AE69-E1C9-4BC8-98CF-FD5498DFC8FC

Figs 3 , 4 , 5

##### Type material.

***Holotype*** (CNHE–11881), 1 ***paratype*** (CNHE–11882), 4 paratypes (CHE–P00146), 1 paratype (HWML–216977).

##### Type host.

*Urobatisjamaicensis* (Cuvier, 1816) (Elasmobranchii: Myliobatiformes: Urotrygonidae).

##### Type locality.

Isla Contoy, Playa Ixmapoit (21°31'44.45"N, 86°48'11.53"W) Quintana Roo, Mexico.

##### Site of infection.

Spiral valve.

##### Prevalence of infection.

14.28%

##### Etymology.

The species is named in honor of M. C. Luis García-Prieto (Universidad Nacional Autónoma de México, Instituto de Biología, México) for his friendship and for his contributions to the knowledge of helminths.

##### Diagnosis.

*Acanthobothriumgarciaprietoi* sp. nov. is a Category 1 species. Body small; relatively robust bothridia; large apical suckers; 7–9 proglottids; long hooks; 18–26 testes per proglottid; cirrus sac and the vagina share a common opening within the genital atrium; arms of ovary symmetrical.

##### Description.

**Figure 3.**
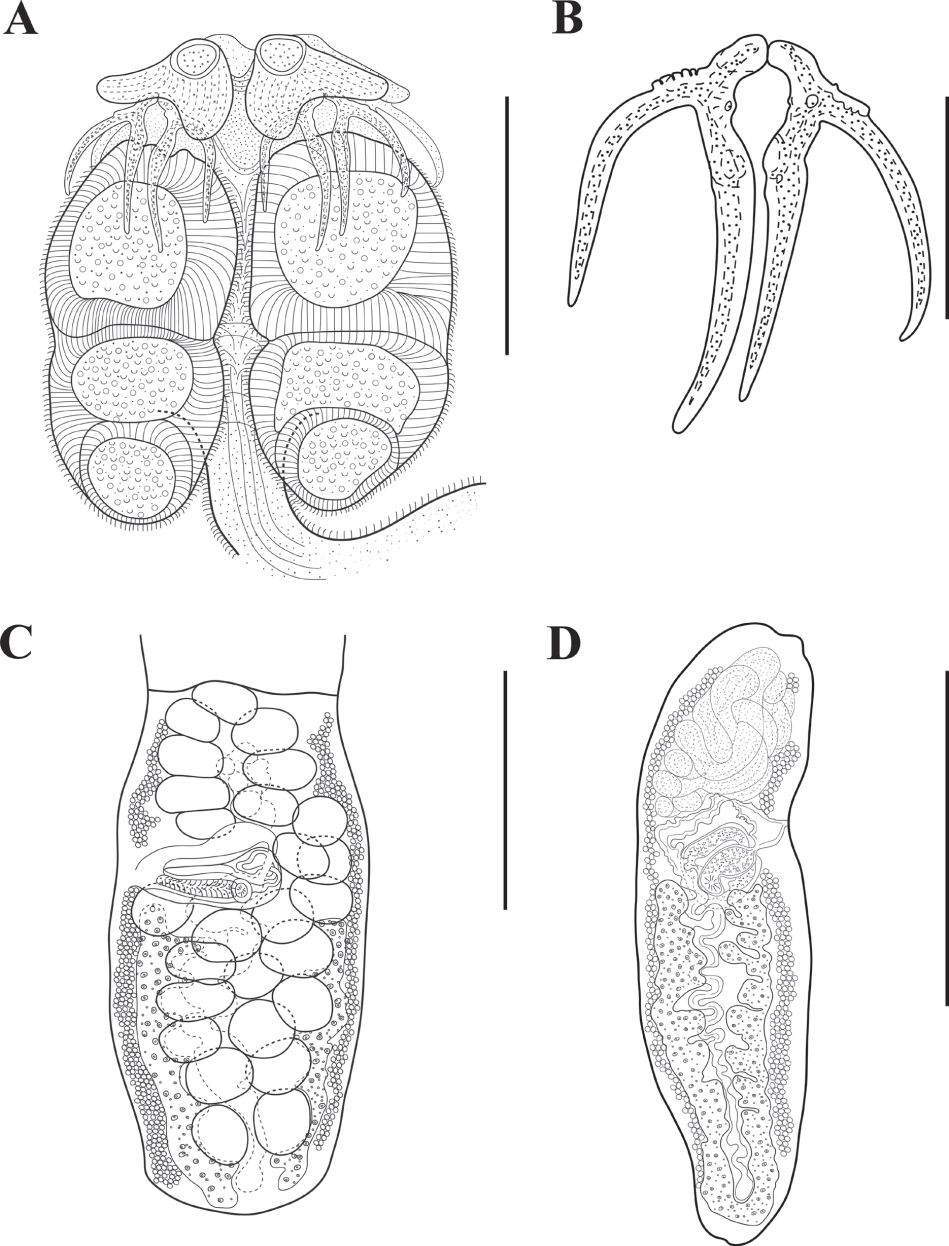
*Acanthobothriumgarciaprietoi* sp. nov. holotype (CNHE–11881). **A** scolex **B** pair of hooks **C** mature proglottid **D** terminal mature proglottid. Scale bars: 70 μm (**B**); 200 μm (**A, C**); 400 μm (**D**).

**Figure 4.**
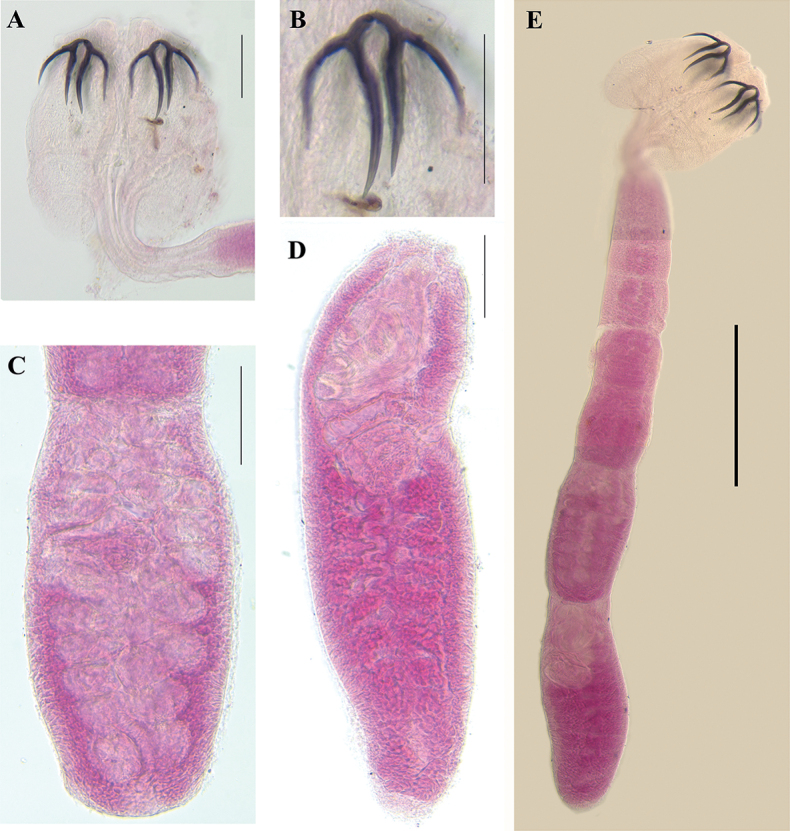
*Acanthobothriumgarciaprietoi* sp. nov. **A–D** holotype (CNHE–11881) **E** paratype (CNHE–11882) **A** scolex **B** pair of hooks **C** mature proglottid **D** terminal mature proglottid **E** complete tapeworm, 400 μm. Scale bars: 100 μm.

**Figure 5.**
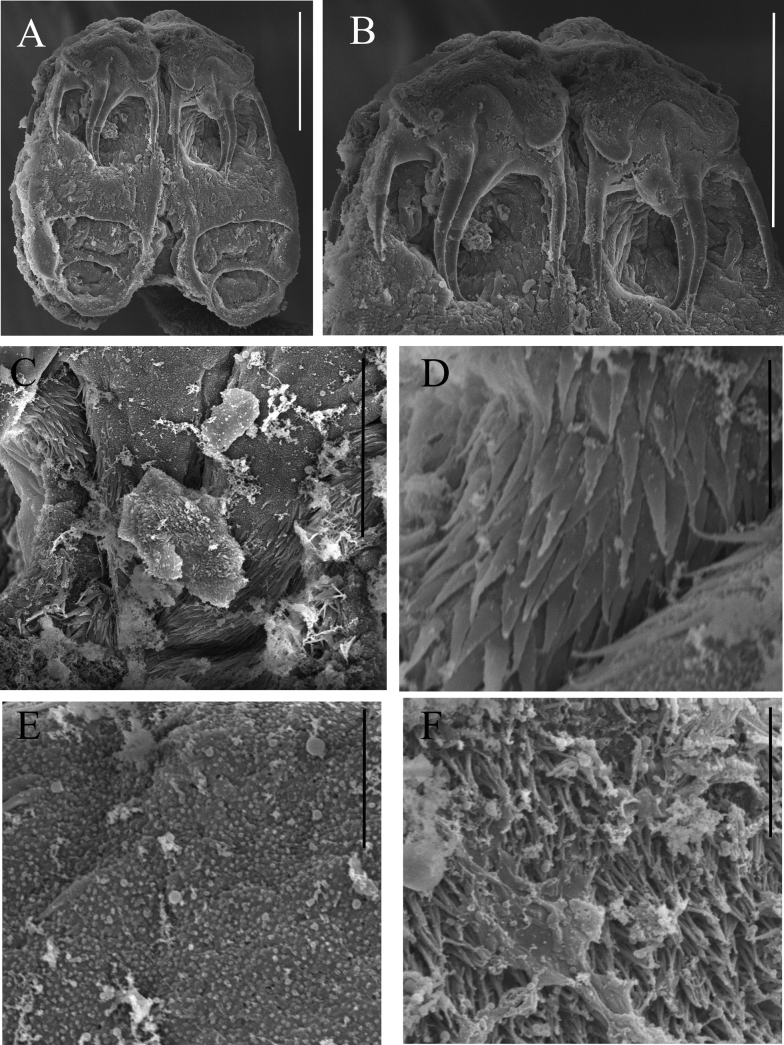
*Acanthobothriumgarciaprietoi* sp. nov. SEM images. Paratype (not deposited) **A** scolex **B** bothridial muscular pad with pair of hooks **C** central area of scolex proper between the bothridia **D** gladiate spinitriches located on the proximal bothridial surface, see image C **E** papilliform filitriches located on the scolex proper, behind the bothridia, see image C **F** coniforme spinitriches located on the cephalic peduncle. Scale bars: 5 μm (**D–F**); 30 μm (**C**); 100 μm (**A, B**).

(Based on 4 complete worms and 9 partial worms mounted on slides.) Entire worms 1.3 mm–2.1 mm (1.8 mm ± 0.4 mm, 4, 4) long; greatest width at scolex, euapolytic; 7–9 (8 ± 1, 4, 4) proglottids per worm (Figs 3, 4). Scolex consisting of scolex proper and cephalic peduncle; scolex proper with four bothridia, 225–320 (278 ± 32; 13, 6) long by 275–323 (305 ± 19; 13, 6) wide; scolex covered with microtriches (gladiate spinitriches and papilliform filitriches, see Chervy 2009) (Fig. 5). Maximum width of scolex at level of middle loculus (Figs 3–5). Bothridia free posteriorly, 245–313 (274 ± 21; 13, 22) long by 135–160 (147 ± 8, 13, 22) wide; each with three loculi separated by two transverse septa, and specialized anterior region in form of muscular pad. Muscular pad 76–83 (79 ± 5, 12, 22) long by 95–110 (101 ± 5, 12, 22) wide, with apical sucker 28–35 (32 ± 3, 12) long by 28–35 (32 ± 2, 12, 22) wide, each with one pair of bipronged hooks at posterior margin (Figs 3–5). Anterior loculus of bothridia 133–168 (154 ± 10, 12, 22) long, middle loculus 50–83 (66 ± 11, 12, 22) long, posterior loculus 50–83 (65 ± 11, 12, 22) long; loculus length ratio 1:0.4:0.4 (anterior:middle:posterior). Velum present between medial margins of bothridia at posterior margin of middle loculus (Figs 3–5).

Hooks bipronged, hollow, with tubercle on the proximal surface of the axial prong; internal channels of axial and abaxial prongs continuous, smooth, the base and anterior part of each hook embedded in muscular pad; axial prongs much longer than abaxial prongs (Figs 3–5). Bases (handles) of medial and lateral hooks articulated with each other; lateral and medial hooks approximately equal in size (Figs 3–5). Lateral hook measurements: A 28–38 (33 ± 2; 14, 25), B 90–105 (96 ± 3; 13, 25), C 78–83 (80 ± 2; 13, 24), D 115–135 (122 ± 6; 13, 24). Medial hook measurements: A’ 25–35 (31 ± 3; 13, 25), B’ 93–110 (102 ± 5; 13, 26), C’ 75–88 (81 ± 3; 13, 26), D’ 115–135 (128 ± 5; 13, 26).

Cephalic peduncle 175–288 (232 ± 35, 13, 7) long by 58–90 (74 ± 11, 13, 7) wide, covered with coniform spinitriches (see Chervy 2009). Proglottids acraspedote. Immature proglottids 133–238 (181 ± 36, 7, 10) long by 93–188 (149 ± 32, n = 10) wide; mature proglottids 260–618 (401 ± 108, 9, 12) long by 133–233 (203 ± 27, 9, 13) wide; terminal mature proglottids 365–550 (480 ± 100, 3, 3) long by 103–205 (169 ± 58, 3, 3) wide (Figs 3–5). Gravid proglottids not observed. Genital pores marginal, irregularly alternating, 26–46% (34 ± 6%, 9, 13) of proglottid length from anterior end in terminal mature proglottids 34–40% (37 ± 3%, 3, 3). Testes oval in dorsoventral view, arranged irregularly anterior to ovarian lobes, two layers deep; testes 30–55 (39 ± 6, 9, 27) long by 50–75 (59 ± 6, 9, 27) wide; testes 18–26 (22 ± 2, 9, 12) in total number, 10–14 (11 ± 1, n = 12) aporal, 3–6 (5 ± 1, 9, 12) preporal, 5–7 (6 ± 1, 9, 12) postporal; no testes located posterior to ovarian isthmus (Figs 3, 4).

Cirrus sac pyriform, 98–143 (125 ± 12, 9, 13) long, 30–88 (50 ± 15, 9, 13) wide in mature proglottids. Cirrus expanded at base, armed with spinitriches. Vagina thick walled, walls covered with gland cells, extending along medial line of proglottid from oötype, reaching to anterior margin of cirrus sac to common genital atrium; vaginal sphincter absent; seminal receptacle not seen. Vagina canal descends on the aporal side of proglottid relatively to the midline of proglottid, often with loops. Ovary with symmetrical lobes; poral lobe 133–375 (241 ± 79, 8, 3) long in mature proglottids, reaching to cirrus sac; aporal lobe 130–375 (240 ± 78, 8, 3) long, extending to the anterior margin of the cirrus sac. Ovary inverted A-shaped in dorsoventral view, lobulated, ovarian isthmus posterior to mid-point of ovary. Mehlis’ gland posterior to ovarian isthmus (Figs 3, 4).

Vitellarium follicular, consisting of two lateral bands; each band consists of two columns of relatively large, elongate to oval follicles, extending from near anterior margin of proglottid to near posterior margin of proglottid, interrupted by vagina and cirrus sac; follicles 345–425 (391± 23, 3, 9) long, 14–22 (17 ± 3, 3, 9) wide. Uterus thin-walled, sacciform, extends from near anterior part of proglottid to near oӧtype. Excretory ducts lateral. Eggs not seen.

##### Remarks.

This is the third species of *Acanthobothrium* described from *U.jamaicensis*. *Acanthobothriumgarciaprietoi* sp. nov. is a category 1 species (sensu Ghoshroy and Caira 2001; Zaragoza-Tapia et al. 2020a): specimens of this species have a total length ≤ 15 mm (worms = 1.3–2.1 mm long); the number of proglottids is ≤ 50 proglottids (the new species has 7–9 proglottids); the number of testes per proglottids is ≤ 80 (the new species has 18–26 testes) and the arms of the ovary are symmetric.

Currently, in Category 1, there are 57 species (Ghoshroy and Caira 2001; Fyler and Caira 2006; Franzese and Ivanov 2020; Zaragoza-Tapia et al. 2020a) but only 12 species inhabit the eastern coast of the Americas: *A.fogeli*, *A.lentiginosum*, *A.lineatum*, *A.schalli*, *A.ulmeri*, and *A.westi* have been reported for the northern Gulf of Mexico; *A.marplatensis*, *A.stefaniae*, *A.carolinae* and *A.domingae* have been reported from the South Atlantic, and *A.cartagenensis*, and *A.himanturi* from the Caribbean Sea.

However, *A.garciaprietoi* sp. nov. should be compared with the species assigned to Category 2 that inhabit the same geographical region because the species in Categories 1 and 2 are similar; the two categories differ only in relation to the symmetry of the ovarian arms. Of the 55 species that are included in Category 2, only two have been reported from the same host and/or to inhabit the same general region: *A.tasajerasi* and *A.urotrygoni*, both from the Caribbean Sea (Table 1).

Of the species of *Acanthobothrium* in category 1, all except *A.stefaniae*, have acraspedote strobili; some are euapolytic or apolytic, and some are hyperapolytic. Members of *Acanthobothrium* known from the Gulf of Mexico are *A.fogeli*, *A.lentiginosum*, *A.lineatum*, *A.schalli*, *A.ulmeri*, and *A.westi*. *Acanthobothriumgarciaprietoi* sp. nov. differ from *A.fogeli*, *A.lineatum*, and *A.schalli* by being shorter, with fewer proglottids, and less testes, except *A.lentiginosum*, *A.ulmeri* and *A.westi*, which overlap in total length, number of proglottids, number of testes and in the diameter of the accessory sucker. *Acanthobothriumlentiginosum* is similar to the new species. However, it has a scolex that, on the average, is longer (288–474 vs. 225–320), and somewhat longer bothridia (272–474 vs. 245–313) with longer loculi; the hooks have a longer abaxial prong (80–110 vs. 78–83), the cephalic peduncle is longer (288–456 vs. 175–288) and the genital pore is differently positioned (59–69 vs. 26–46, respectively). *Acanthobothriumulmeri* differs from the new species by having a larger scolex (357–464 vs. 225–320), longer bothridia (281–432 vs. 245–313) and larger loculi; the hooks are shorter, with the axial prong (61–78 vs. 90–105) long and the abaxial prong (59–72 vs. 78–83) long; prongs that are both shorter in total length (77–103 vs. 115–135), and the cephalic peduncle is shorter (48–176 vs. 175–288). *Acanthobothriumwesti* differs from the new species in having the scolex and smaller loculi (215–277 vs. 225–320, respectively), hooks with shored handles (25–28 vs. 28–38), axial prongs (42–53 vs. 90–105), and the abaxial prongs (38–48 vs. 78–83); the cephalic peduncle is longer (216–480 vs. 175–288), the cirrus sac is shorter (72–84 vs. 98–143), and range in the number of testes is less (20–24 vs. 18–26).

Of the species of *Acanthobothrium* reported from the South Atlantic, *A.marplatensis*, is much larger (4.79 mm–8.44 mm vs. 1.3 mm–2.1 mm for the new species), it has a greater number of proglottids (18–30 vs. 7–9, respectively), more testes per proglottid (24–39 vs. 18–26). The hooks of *A.marplatensis* are shorter, the cirrus sac rises towards the anterior end of the proglottid and the cirrus sac of the new species extends horizontally in mature proglottids (Fig. 3C) or slightly posterior in some terminal proglottids. *Acanthobothriumstefaniae* has craspedote proglottids and is hyperapolytic; it has a smaller scolex (285–400 vs. 225–320), somewhat longer bothridia (247–355 vs. 245–313) and longer anterior loculi; the handles of the hooks are relatively shorter (25–32 vs. 28–38), the abaxial prongs are shorter (32–52 vs. 78–83), the axial prong is longer (102–112 vs. 90–105). In *A.stefaniae*, the genital pore opens at approximately mid-proglottid and, in *A.garciaprietoi* sp. nov., it opens at the anterior third of the proglottid.

*Acanthobothriumcartagenensis* and *A.himanturi* have been reported from the Caribbean Sea. *Acanthobothriumcartagenensis* differs from and *A.garciaprietoi* sp. nov. by having a greater number of proglottids (13 vs. 7–9, respectively); its scolex is square (300–300), while that of *A.garciaprietoi* sp. nov. is wider than long (225–320 long 275–323 wide); the loculi of *A.cartagenensis* are shorter (1:0.3:0.3 vs. 1:0.4:0.4). The apical pads of *A.cartagenensis* are 67 in diameter, and they are relatively elliptical with no invaginations in the hook region; in contrast, those of *A.garciaprietoi* sp. nov. are larger (76–83 long by 95–110 wide), with an invagination in the region between the hooks. The cephalic peduncle of *A.caragenensis* is shorter than that of *A.garciaprietoi* sp. nov. (180 long by 120 wide vs. 175–288 long by 58–90 wide). The testes of *A.cartagenensis* are smaller in diameter (30–45 vs. 50–75 long by 30–55 wide of *A.garciaprietoi* sp. nov.), but the cirrus sac of *A.garciaprietoi* sp. nov. is larger. The lobes of the ovary of *A.cartagenensis* are longer than those of *A.garciaprietoi* sp. nov. (210–390 long by 60–112 wide vs. 133–375 long).

*Acanthobothriumhimanturi* differs from the new species by being longer (3.84–9.3 mm vs. 1.3–2.1 mm, respectively), slightly large scolex (240–350 vs. 225–320), longer bothridia (297–432 vs. 245–313) and longer cephalic peduncle (300–1,000 vs. 175–288). The handles of the hooks of *A.himanturi* are 43–61 long, the abaxial prong is 73–99 long, and the axial prong is 86–116 long, all being longer (28–38, 78–83, 90–105, respectively) than those of *A.garciaprietoi* sp. nov. It has a greater number of testes (38–57 vs. 18–26) and the ovarian lobes are longer and narrower (480–600 vs. 133–375) than those of *A.garciaprietoi* sp. nov.

Compared to the other two species in Categories 1 and 2, *A.garciaprietoi* sp. nov. differ from *A.carolinae* by the shorter body (1.3 mm–2.1 mm vs. 1.81mm–3.93, respectively), smaller number of proglottids (7–9 vs. 8–17), and the larger hooks. The new species *A.garciaprietoi* sp. nov., differs from *A.domingae* by the body size (1.3 mm –2.1 mm vs. 3.5 mm –5.4 mm, respectively), the number of proglottids (7–9 vs. 14–18, respectively), and the smaller hooks (115–135 vs. 168–238, respectively).

## ﻿Discussion

It is good practice to describe a species based on as large number of specimens as possible and, if the material permits, include molecular information on the species. However, sometimes the material is limited and, in name of “the progress of knowledge and science” (Thomson et al. 2018), this sub-representation is accepted because the alternative is to fail to recognize new aspects of biodiversity. Recent examples are the descriptions of species of *Acanthobothrium* by Caira and Burge (2001), Campbell and Beveridge (2002), Fyler and Caira (2010), Maleki, Malek, and Palm (2015), and Gallagher and Caira (2020). The entire coast of Quintana Roo now is an Área Natural Protegida (SEMARNAT 2018), making it difficult to collect new material that would augment the samples collected more than 23 years ago. Of course, if future circumstances permit, it is advisable to supplement these specimens with freshly-collected specimens.

### ﻿Knowledge of helminths in elasmobranchs

The study of parasites of elasmobranchs in Mexico was initiated by Caballero y Caballero (1945) (Merlo-Serna and García-Prieto 2016). Later, Winter (1959) and then Bravo-Hollis (1969) made important contributions by describing new species of Digenea and Monogenea. More recently, the knowledge of the diversity of the helminths of elasmobranchs in Mexico has been increased by the descriptions of new species and new records of monogeneans by Pulido-Flores and Monks (2005, 2008, 2014), Pulido-Flores et al. (2015), and Quiterio-Rendon et al. (2018). For Digenea, Rodríguez-Ibarra et al. (2011) and Zaragoza-Tapia et al. (2013). For Cestoda, Caira (1985a), Monks et al. (1996), Caira and Zahner (2001), Caira and Bruge (2001), Ghoshroy and Caira (2001), Monks et al. (2015b), Monks et al. (2015a), Monks et al. (2015c), Rodríguez-Ibarra et al. (2018), Zaragoza-Tapia et al. (2013), Zaragoza-Tapia et al. (2019), and Zaragoza-Tapia et al. (2020b) contributed new data.

As can be seen from the cited works, the majority of the species from elasmobranchs that have been described to date from Mexican waters are cestodes. Many of them have been assigned to the genus *Acanthobothrium*, which currently is represented world-wide by 213 nominal species. Of these, 25 have been recently described: 13 from the Atlantic (Franzese and Ivanov 2018, 2020; Rodríguez-Ibarra et al. 2018; Van Der Spuy et al. 2020; Van Der Spuy et al. 2022; this study), six from the Persian Gulf, Gulf of Oman, and Indic Ocean (Maleki et al. 2018, 2019), and six from the Pacific Ocean (Zaragoza-Tapia et al. 2019, 2020b; Gallagher and Caira 2020). Table 2 includes the most recently-described species as a complement to the information presented by Zaragoza-Tapia et al. (2020a). These data are integrated into the original table from that work (Suppl. material 1). The geographical distribution of the known localities of cestodes of elasmobranchs continues to be very unequal, representing the geographical distribution of the taxonomists that described the species rather than the expected global distributions of members of the order (Zaragoza-Tapia et al. 2020a).

### ﻿Species with a “clover leaf” configuration of the scolex

The majority of species of *Acanthobothrium* have scolex moderately to completely attached to the scolex proper by membranous tissue (velum) that holds the bothridia close to the scolex. In contrast, the species with a “clover-leaf” scolex (Yoshida 1917) have bothridia either completely unattached along almost the entire length or attached only by a long, tenuous velum membrane. This allows the bothridia to project laterally from the anterior-most part of the bothridia, thus forming a laterally-wide surface (Figs 1A, 2A) used to attach to the intestinal wall of the host. This configuration has been called “clover leaf” for a general resemblance to the leaf pattern of the clover plant (*Trifolium* Linnaeus), which commonly has 3–5 leaves attached around the terminal point of the stem. Some authors (e.g., Yoshida 1917; Fyler and Caira 2010) have considered this conformation of the scolex to be a valuable morphological character to differentiate a number of species. However, this type of conformation suggests three possibilities: an adaptive change to increase the area of bothridial attachment in a monophyletic group; a parallel evolution of a similar function in species not closely related; or secondary functional increase in bothridial attachment as a result of lengthening of the velum allowing better movement of the bothridia. Since the length of the velum is variable within *Acanthobothrium* spp. and no phylogenetic hypothesis for all putative species exists, either is possible or it might be the result of some other mechanism.

Within *Acanthobothrium*, 13 species from Australian, the Western Pacific region, and Indian Ocean were described as having a “clover-leaf” scolex: *A.adlardi*, *A.cestraciontis*, *A.dasybati*, *A.grandiceps*, *A.karachiense*, *A.ijimai*, *A.macrocephalum*, *A.mujibi*, *A.pingtanensis*, and *A.robertsoni*. Three other species (*A.majumdari*, *A.tsingtaoensis*, and *A.zugeinensis*) are not included in the list above because they are not considered valid taxa by Caira et al. (2012) and Caira and Jensen (2017). The latter authors categorized them as species *inquirendae*. These species were included in the comparisons with *A.pulidofloresae* sp. nov. using the data recorded in the original descriptions because they were described as having a “clover-leaf” scolex, described as reminding a “four-leaved clover” or drawn as having this conformation. All of the aforementioned species occur in the greater Pacific Basin. In contrast, *A.pulidofloresae* sp. nov. occurs in the Caribbean. How this interpretation of the distribution awaits a phylogenetic hypothesis for the members of the genus.

## Supplementary Material

XML Treatment for Acanthobothrium

XML Treatment for Acanthobothrium pulidofloresae

XML Treatment for Acanthobothrium garciaprietoi

## ﻿Additional information

### Author ORCIDs

Erick Rodríguez-Ibarra https://orcid.org/0000-0002-2930-8709

Juan Violante-González https://orcid.org/0000-0001-6291-8763

Scott Monks https://orcid.org/0000-0002-5041-8582

### Data availability

All of the data that support the findings of this study are available in the main text or Supplementary Information.

## Supplementary materials

Supplementary material 1 Update to Zaragoza-Tapia et al. 2020- Host relationships and geographic distribution of species of *Acanthobothrium* Blanchard, 1848 (Onchoproteocephalidea, Onchobothriidae) in elasmobranchs: a metadata analysis (up to 2020 to 2022) Data type Occurrences and distribution Explanation note This document is an update of the original data set of the host relationships and biographic distribution of parasites of the genus *Acanthobothrium* of the elasmobranch definitive host, to include species described from 2020 to 2022. https://binary.pensoft.net/file/877631This dataset is made available under the Open Database License (http://opendatacommons.org/licenses/odbl/1.0/). The Open Database License (ODbL) is a license agreement intended to allow users to freely share, modify, and use this Dataset while maintaining this same freedom for others, provided that the original source and author(s) are credited.Erick Rodríguez-Ibarra, Juan Violante-González, Scott Monks
